# Flow rate resonance of actively deforming particles

**DOI:** 10.1038/s41598-023-36182-5

**Published:** 2023-06-10

**Authors:** Daniel R. Parisi, Lucas E. Wiebke, Judith N. Mandl, Johannes Textor

**Affiliations:** 1 Instituto Tecnológico de Buenos Aires (ITBA), CONICET, C.A. de Buenos Aires, Argentina; 2 Instituto Tecnológico de Buenos Aires (ITBA), C.A. de Buenos Aires, Argentina; 3 Department of Physiology and McGill Research Centre on Complex Traits, McGill University, Montreal, Canada; 4 Data Science group, Institute for Computing and Information Sciences, Radboud University, Nijmegen, The Netherlands

**Keywords:** Biological physics, Computational biophysics

## Abstract

Lymphoid organs are unusual multicellular tissues: they are densely packed, but the lymphocytes trafficking through them are actively moving. We hypothesize that the intriguing ability of lymphocytes to avoid jamming and clogging is in part attributable to the dynamic shape changes that cells undergo when they move. In this work, we test this hypothesis by investigating an idealized system, namely, the flow of self-propelled, oscillating particles passing through a narrow constriction in two dimensions (2D), using numerical simulations. We found that deformation allows particles with these properties to flow through a narrow constriction in conditions when non-deformable particles would not be able to do so. Such a flowing state requires the amplitude and frequency of oscillations to exceed threshold values. Moreover, a resonance leading to the maximum flow rate was found when the oscillation frequency matched the natural frequency of the particle related to its elastic stiffness. To our knowledge, this phenomenon has not been described previously. Our findings could have important implications for understanding and controlling flow in a variety of systems in addition to lymphoid organs, such as granular flows subjected to vibration.

## Introduction

The flow of densely crowded particles through narrow constrictions is a key scenario in the active matter field that has been widely studied in a range of experimental and theoretical models, motivated, for instance, by the need to evacuate pedestrians from a room quickly and safely in case of an emergency^1–3^ or by the need to avoid grains being stuck when flowing out from a silo^4^. More recently, cell biologists have begun to apply concepts such as jamming and fluid-to-solid transitions to the study of dense multicellular tissues^5,6^. Viewed through the prism of active matter research, the study of dense multicellular systems raises a number of intriguing questions, given that cells have some key properties that set them apart from other self-propelled agents: they move at very low Reynolds numbers and achieve their motion by dynamically changing their shape to generate friction and traction forces. Among the body’s many types of cells, T cells^7^, neutrophils^8^, and other types of immune cells are the champions in regard to motility: they can enter and move within almost any tissue. Immune cells also often move in large crowds. T cells searching for a foreign antigen, for instance, form large, dense collectives of millions of cells^9^. Other examples of motile cell collectives are found during morphogenesis and wound healing^10^. Therefore, the study of migrating cell crowds might produce important insights into how to keep active matter systems in a fluid state in challenging conditions.

The biophysics of cell migration are complex and involve interacting networks of actins, myosins, and other molecules. However, a commonality of all cells is that they need to change their shape to move. There are different types of shape changes as in *lamellopodia*^11^, broad extensions at the front of the cell, or *blebs*^12^, pressure-driven and spherical expansions of the plasma membrane.

These special properties of cell migration motivate us to study the influence of deformations on particle flow through a narrow constriction. As a starting point, we consider the simplest possible system with deformable agents: two-dimensional (2D) circular particles that interact via linear elastic forces with friction and actively change their radii while moving. Although cells maintain an approximately constant volume during movement, the area of contact with the substrate can change significantly. Therefore, a 2D representation of a particle changing its radius can be considered a projection of the cell’s 3D shape onto the motion plane. Nevertheless, we note that our intention is not to propose a realistic model of cell migration but rather to investigate the fundamental impact of deformation in an idealized flow-clogging system.

Previous studies have investigated the bulk properties of idealized active matter systems with shape-changing particles^10,13,14^. Our model is inspired by the work of Tjhung and Berthier^13^, who simulated actively deforming circular particles that contract and expand their radii sinusoidally while confined into a closed environment. They found that as a consequence of these area fluctuations, a phase transition to collective motion emerges beyond a critical amplitude. Building upon their work, we investigate the effects of active deformation in a very different scenario: self-propelled particles flowing through a narrow constriction.

Our simulations support the idea that active deformations can help prevent detrimental crowding effects: we observe a transition from clogging to flowing with respect to the amplitude but also with respect to the frequency. Moreover, we find an intermediate and optimum frequency ($w^*$) value that maximizes the flow rate.

## Numerical simulations

### Model

Our proposed model comprises particles that are active in two ways: (a) each particle is subject to a driving force attempting to move it towards a specific goal, and (b) each particle undergoes active deformation through harmonic oscillation of its radius. Additionally, the particles interact upon contact with each other and the boundaries.

The driving and contact forces utilized in our model are derived from the Helbing-Farkas-Vicsek model^2^. As a result, the equation of motion for each particle *i* can be expressed using Eq. (1):

1 $$\begin{aligned} m_i \frac{d{\textbf{v}}_i}{dt} = {\textbf{F}}_{di} + {\textbf{F}}_{ci} \end{aligned}$$

where $m_i$ is the mass of particle *i* and ${\textbf{v}}_i$ is its velocity. The driving force (${\textbf{F}}_{di}$) of the active mechanism by which the particle moves is given by Eq.  (2):

2 $$\begin{aligned} {\textbf{F}}_{di} = m_i \frac{v_{fi} {\textbf{e}}_{di} - {\textbf{v}}_i}{\tau } \end{aligned}$$

where $v_{fi}$ is the free velocity magnitude at which the particle moves in the absence of contacts, ${\textbf{e}}_{di}$ is the unit vector pointing towards the destination, and $\tau$ is a relaxation constant that can be interpreted as the time needed for a particle, initially at rest, to achieve its final speed $v_{fi}$.

**Figure 1:**
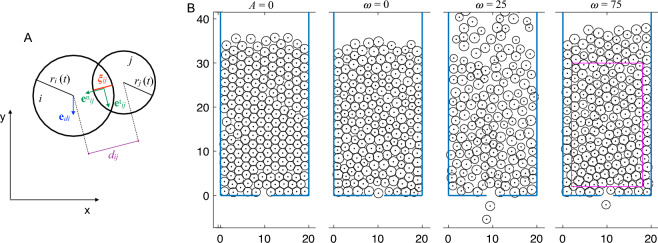
Model and simulated system definition. A: Geometrical quantities of two interacting particles. B: Lower part of the simulated box with an opening at $y=0$. Four different conditions are shown. For $\omega$=0, 25, and 75, the amplitude is set to $A=0.15$. The rest of the parameters correspond to the reference system (see text). The magenta lines shown in panel B, $\omega = 75$ define the measurement area used in the results section.

The contact force exerted upon particle *i* by its contacting neighbours *j* and contacting boundaries *b* incorporates normal repulsion and tangential friction and is computed by Eq.  (3):

3 $$\begin{aligned} {\textbf{F}}_{ci} = \sum _{j}g(\xi _{ij})~\lbrace ~(k_n~{\textbf{e}}^n_{ij}) ~ + ~ (k_t~[({\textbf{v}}_j-{\textbf{v}}_i). {\textbf{e}}^t_{ij}] ~{\textbf{e}}^t_{ij})~\rbrace + \sum _{b}g(\xi _{ib})~\lbrace ~(k_n~{\textbf{e}}^n_{ib}) ~ + ~ (k_t~[-{\textbf{v}}_i. {\textbf{e}}^t_{ib}] ~{\textbf{e}}^t_{ib})~\rbrace \end{aligned}$$

where $k_n$ and $k_t$ are the normal and tangential stiffness coefficients, respectively, and the following geometrical quantities are displayed in Fig. 1A: $\xi _{ij}$ is the overlap defined as $\xi _{ij} = (r_i + r_j) - d_{ij}$, where *r* is the radius, $d_{ij}=|{\textbf{x}}_{i}-{\textbf{x}}_{j}|$ is the distance between particle centres, and ${\textbf{x}}$ denotes the positions of the particles; *g*(*z*) is a function that is zero when $z<0$ (particles are not in contact if $\xi _{ij}<0$) and equal to the argument *z* otherwise; ${\textbf{e}}^n_{ij}$ is the normal unit vector ${\textbf{e}}^n_{ij}=(e^{n1}_{ij},e^{n2}_{ij})=\frac{{\textbf{x}}_{i}-{\textbf{x}}_{i}}{d_{ij}}$; and ${e}^t_{ij}=(-e^{n2}_{ij},e^{n1}_{ij})$ is the tangential direction at the contact point. The second sum of Eq.  (3) corresponds to particles overlapping with one or two boundaries and uses similarly defined quantities.

The other important ingredient of the present system is the active deformation represented by the forced oscillating radius according to Eq. (4):

4 $$\begin{aligned} r_i(t) = r_0 ~ (1 + A ~ \text {sin}(\omega t + \phi _i ) ) \end{aligned}$$

where $r_0$ is the radius at rest, which is equal for all particles, *A* is the amplitude of the harmonic oscillation, and $\phi _i$ is a random phase for each particle. Note that in the case of $A=0$, the radii are constant and equal for all particles forming a monodisperse system. In the case of $\omega =0$, the radii are constant (non-oscillating) but differ between particles due to the individual random phases, forming a polydisperse system.

### Simulated system

As previously stated, we investigate a 2D system comprising self-propelled and self-deforming disks. To maintain generality, we employ dimensionless units, where the mass of all particles is set to $m=1$, the resting radii are set to $r_0=1$, and the relaxation constant is set to $\tau =1$. For instance, a distance expressed as $d_{ij}=3$ corresponds to a distance of $3r_0$, and a simulation time of $T=5000$ corresponds to $5000\tau$.

The remaining parameters of the reference system are set as follows: $k_n=500$, $k_t=1000$, the number of particles $N=200$, and $v_f=3.3$. The variables under investigation are $A \in [0, 0.3]$ and $\omega \in [0, 100]$. For specific studies, the following parameters are perturbed: $v_f \in [1, 10]$, and $k_n \in [150, 1500]$ (thus, $k_t= 2 \times k_n$). To keep the friction force approximately constant, we maintain the $k_n/k_t$ ratio constant since the overlap decreases as the value of $k_n$ increases. This is because the friction force is dependent on the overlap, as shown in Eq. (3).

Figure 1B illustrates the geometry of our system, which is a rectangular box with dimensions of 20 by 150 and features a constriction at the bottom with a width of $D=2.8$. The value of *D* is chosen within the range where intermittent or clogged flow occurs, i.e., within a few particle radii, as smaller values of *D* can completely block the flow, and larger values lead to the disappearance of clogging effects^15^. We initialize the particles by placing them randomly inside the container, ensuring that they do not overlap with boundaries or other particles. We accomplish this by rejection sampling, i.e., by creating the first particle at a random position one radius away from any boundary and subsequently generating each $i^{th}$ particle (for $i=2,...,N$) randomly. If the new particle overlaps with an existing particle *j* ($\xi _{ij}>0$, see Eq. (3)), it is discarded. However, if there is no overlap ($\xi _{ij}\le 0$), the particle is considered valid. This process iterates until $i=N$. Note that initially, the particles are not in contact (i.e., not packed).

While the system is flowing, particles that reach $y=-15$ are reintroduced at a random position in the ranges $2<x<18$ and $100<y<150$, again avoiding overlaps by rejection sampling. This conservation of particle number ensures that, after a transient burn-in period, the stationary regime is obtained.

To avoid artefacts resulting from arbitrary heuristics at the exit, we set the destination of all particles to be constant in the $-y$ direction ( ${\textbf{e}}_{di}=(0,-1)$ ), as illustrated in Fig. 1A. Importantly, this approach only fixes the preferential direction, as the driving force vector varies according to Eq.  (2).

The equations of motion for all particles, as given by Eq.  (1), are integrated using the Verlet algorithm with a time step of $dt=10^{-4}$. The positions, velocities, and radii of all particles are recorded at every time step $t=t_k$ with intervals of $dt_k=0.5$. Typical simulation times *T* range from 5000 to 25,000.

## Results

In this section, we focus on examining how the system responds to changes in the amplitude (*A*) and frequency ($\omega$) of self-oscillation using the set of reference parameters defined in the previous section.

### Self-oscillation amplitude

Here, we analyse the system’s behaviour as we vary the oscillation amplitude while keeping the frequency fixed at $\omega = 20$.

**Figure 2:**
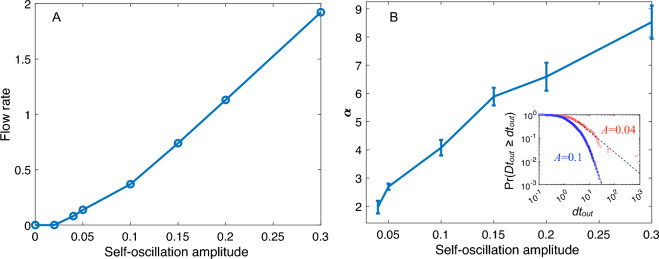
Flow characterization as a function of the self-oscillation amplitude. A: Flow rate; 95% confidence intervals are within the symbol size. B: The $\alpha$ exponents of the power-law distribution of the time intervals between two consecutive particles passing through the exit ($dt_{out}$). In the inset, two examples of complementary cumulative distribution functions (also known as survival curves) are shown from which $\alpha$ is estimated^19^. The y-axis label indicates the probability of finding any passage time $Dt_{out}$ greater than a particular value of $dt_{out}$. The dashed lines indicate the tail of the distributions where the power law is valid ( $dt_c: dt_{out} \ge dt_{out}^{min}$ ).

Figure 2A shows that the flow rate monotonically increases with increasing amplitude. This behaviour is expected due to the increasing energy of the local fluctuations. This is also in accordance with previous reports on vibrated silos where inert granular material flows through the bottom opening^15–18^.

Furthermore, it can be observed that the system does not flow at all for very low amplitudes. Specifically, in the case of $A=0$, the system is monodisperse, where all radii are equal ($r_i = r_0$, for $i = 1,..., N$). Because the exit is only 40% larger than one particle diameter, it can be easily blocked, causing the flow to stop.

Similar to the case of actively deforming particles without a driving force^13^, there exists a critical amplitude at which the system starts to show net displacement of particles and flow occurs out of the container. Under the present conditions, this amplitude is approximately $A=0.04$. However, strictly speaking, even at $A=0.04$, the system can still be considered clogged, based on the definition of the *flowing parameter* ($\Phi$) introduced by Zuriguel et al.^15^. This parameter allows for the discrimination between clogged ($\Phi =0$), intermittent ($0< \Phi < 1$), and flowing ($\Phi =1$) states of particles passing through a narrow constriction and is mathematically defined as $\Phi =\langle dt_f \rangle / (\langle dt_c \rangle + \langle dt_f \rangle )$, where $\langle dt_f \rangle$ represents the average duration of the bursts (continuous flow periods), and $\langle dt_c \rangle$ represents the average clog duration (arrested periods). There is substantial evidence in the literature that discrete flows of particles with interactions similar to those in the present study follow exponential distributions for burst ($dt_f$)^15,20–23^, and power-law distributions for clogging times ($dt_c$)^15,16,24–28^.

In the case of an exponential distribution, the average is always well defined, so $\langle dt_f \rangle$ is a finite number. On the other hand, the power-law distribution $dt_c^{-\alpha }$ has only a finite mean value if $\alpha > 2$, which leads to $\Phi > 0$. If $\alpha \le 2~\Rightarrow ~\langle dt_c \rangle ~\rightarrow ~\infty$, then $\Phi = 0$, indicating that the system is clogged even if it displays some flowing periods (as seen in Fig. 2 A where the flow rate of $A=0.04$, calculated during a finite simulation time, is greater than zero). In other words, an exponent $\alpha \le 2$ indicates that in the limit of infinitely long experiments, the ratio between flowing times and clogging times tends to zero ($\Phi =0$).

To calculate the exponent $\alpha$, we define $dt_{out}$ as the time between consecutive exiting particles, regardless of whether they belong to a flowing or clogging period. We then utilize the Clauset-Shalizi-Newman method^19^ to analyse the survival curve of $dt_{out}$ and determine the value of $\alpha$, as well as $dt_{out}^{min}$, which represents the minimum value of $dt_{out}$ for which the data display power-law behaviour. This criterion can be used to define when a clog event occurs ($dt_c \ge dt_{out}^{min}$), and therefore, the flowing period $t_f$ occurs between two clogging events. The inset of Fig. 2(B) shows the results of these analyses, indicating that the case of $A=0.04$ has a much greater probability of developing long-lasting clogs ($\alpha \sim 2$) than the case of $A=0.1$ (which has a greater $\alpha$ value).

If we call the duration of a simulation *T*, by definition, $T=\sum dt_{out}$. Considering that the mean flow rate *Q* is the number of particles exiting during that period ($Q=N/T$), we obtain $Q=N/\sum dt_{out}=1/\langle dt_{out} \rangle$. Thus, because distributions of $dt_{out}$ with higher values of $\alpha$ correspond to lower values of $t_c$, the average $\langle dt_{out} \rangle$ is lower, and the flow rate is higher. This explains why the increase in the flow rate *Q* is associated with the increase in the $\alpha$ exponent, as observed in Figure 2.

Beyond the amplitude $A=0.04$, it can be observed in Fig. 2B that the $\alpha$ exponents are greater than 2, indicating that the system is in flowing states.

In summary, it is worth highlighting that despite the difference between a local vibration caused by the active deformation of particles and a bulk external vibration of the granular material container, both types of vibrations yield the same outcome. Specifically, increasing the vibration amplitude enhances the flow and leads to a transition from a clogged to an unclogged phase^15^.

### Self-oscillation frequency

We now shift our focus to various observables that are influenced by changes in the frequency of active deformation of particles. For this purpose, we set the amplitude to moderate values ($A = {0.10, 0.15}$). The other parameters not explicitly mentioned here are kept at their reference values as defined in the “Simulated System” section. As mentioned before, even at zero frequency, the system always results in a polydisperse particle system due to random phases. More precisely, the particle radii are generated at $t=0$ according to a distribution that is determined by the amplitude *A*.

**Figure 3:**
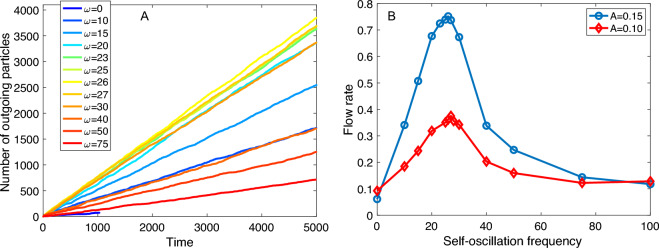
Flow characterization for variable self-oscillation frequencies. A: Examples of the first $t=5000$ of the discharge curves for $A=0.15$. The flow rates are calculated from the complete curves. B: Flow rate as a function of $\omega$.

Considering the amplitude $A=0.15$, Fig. 3A shows the discharge curves for different values of $\omega$. The flow rate was calculated from the slopes of these curves by linear interpolation using the “regress” function from MATLAB, which provides 95% confidence intervals for the coefficient estimates. It can be observed that for $\omega =0$, the discharge curve is interrupted before reaching the end of the simulation time, indicating flow arrest due to clogging. For this reason, the number of exiting events is very low and not sufficient to compute the $\alpha$ exponent. Therefore, we conducted five simulations with different initial conditions, all of which resulted in an arrested flow, mostly before reaching $t=1000$. These simulations allowed us to record 388 exiting events, from which we calculated the power-law exponent. The obtained value of $\alpha =1.9 \pm 0.2$ suggests that $\omega =0$ may correspond to a clogged state in practical terms. However, the system exhibits intermittent and flowing states for the frequencies above $\omega =0$ that we have studied. It is worth noting that for higher $v_f$ values, we observe the transition from clogging to flowing states at higher $\omega$ thresholds.

The flow rate as a function of the frequency is displayed in Fig. 3B for $A=0.10$ and $A=0.15$. Surprisingly, we observe an optimum frequency ($\omega ^* \sim 25$) at which the flow rate is maximized for both amplitudes.

Continuing our analysis, we aim to gain a better understanding of why the system exhibits the maximum flow rate at a particular frequency. There are at least two possible explanations for this behaviour.

First, it could be a local effect related to the time scale needed for a particle to pass through the exit orifice. The period $t=2 \pi /\omega ^* \sim 0.24$ is the approximate expected time a particle takes to cross this exit, which is at least on the order of $t' = r_o/v_f = 1/3.3 = 0.3$.

Second, it could be a global effect related to the natural frequency of the system ($\omega _0$). Since our simulated particles have linear interactions primarily determined by the normal stiffness coefficient $k_n$, we expect that the square root of $k_n$ is related to the natural frequency as follows: $\omega _0 = (k_n/m)^\gamma$, with $\gamma =0.5$, and *m* is the particle mass, which we set to $m=1$. As a first estimation, the optimum frequency $\omega ^*=25 \pm 3$ is similar to the square root of the normal stiffness coefficient $\sqrt{k_n/m} = \sqrt{k_n} = \sqrt{500} \sim 22$.

**Figure 4:**
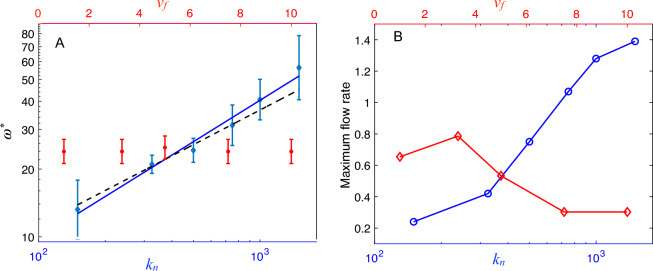
Optimal active frequency ($\omega ^*$) and the corresponding maximum flow rate as a function of the free speed ($v_f$, linear, red, top x-axis) and the stiffness coefficient ($k_n$, logarithmic, blue, bottom x-axis). When $k_n$ is varied, the value of the free velocity is fixed at $v_f=3.3$. When $v_f$ is varied, the value of $k_n$ is fixed at $k_n=500$. A: Optimal frequencies that maximize the flow rate. The simulated data for varying $k_n$ are fitted with the proposed relation $\omega ^*=k_n^{\gamma }$, with $\gamma =0.6\pm 0.1$ (blue, solid line) and with $\omega ^*=~1.2~\sqrt{k_n}$ (black, dashed line). See Supplementary Information for details on the error bar estimates. B: Maximum flow rate corresponding to each point of $\omega ^*$ from Panel A.

To determine which explanation is correct, we conducted studies similar to those shown in Fig. 3B to identify the optimum frequency, $w^*$, for different values of the free velocity, $v_f$, and the normal stiffness coefficient, $k_n$ (see Supplementary Information). As shown in Fig. 4A, we found that the optimum frequency remained constant for varying $v_f$, but $w^*$ did depend on the value of $k_n$. The log-log plot of this relationship indicates that it can be approximated by a power law function $w^* = k_n^{\gamma }$, where the exponent is found to be $\gamma = 0.6 \pm 0.1$ or by $w^* = 1.2~ \sqrt{k_n}$. These findings provide evidence supporting that the optimum frequency that maximizes the flow is related to the natural frequency of particles ($\sqrt{k_n}$). To complete the view, Fig. 4B shows the peak flow rate values corresponding to the optimum frequencies. It can be seen that the flow rate exhibits a maximum and then decreases for increasing values of the free velocity, in accordance with the faster-is-slower effect^2,15,25^. Additionally, an increase in the flow rate is observed for higher values of $k_n$. This is a novel effect due to the active oscillation of particles, which is not observed in flows of rigid particles.

The observation that the flow rate reaches a maximum when the self-oscillation frequency of particles is similar to the natural oscillation of the linear interaction is consistent with the theory of the driven harmonic oscillator. When an external harmonic force with frequency $\omega$ (in our case, the active self-oscillation of particles) is applied to a spring (in our case, the particle’s interaction given by $k_n$), the resulting oscillation of the spring has an amplitude ($A_0$)^29^ that is proportional to $A_0 \propto 1 / \sqrt{(2 \omega \omega _0 \zeta )^2 +(\omega ^2-\omega _0~^2)^2}$, where $\zeta$ is the damping ratio. This implies that a single resonance peak is produced and that the amplitude tends to zero as the value of $\omega$ approaches infinity. Therefore, as the active frequency increases, if two particles come into contact, the resulting amplitude of the interaction approaches zero. In other words, a very rapid change in the particle radii results in an effective radius $r_{eff} (t) \sim r_0$, as if it were not fluctuating at all. This picture is consistent with Fig. 3B, where the flow rate for large values of $\omega$ is similar to that of $\omega =0$.

#### Factors contributing to the maximum flow rate

To obtain additional insights, we focused on the reference system with $k_n=500$, $v_f=3.3$, and $A=0.15$ (already analysed in Fig. 3) and investigated observables that quantify the frequency and intensity of particle overlaps as a function of $\omega$. These measurements were taken in a bulk region defined by $x \in [2, 18]$ and $y \in [2, 30]$ (see illustration in Fig. 1B for the case of $\omega =75$).

We examine the probability of finding a particle inside this area that is free of contact from any other particle, which we refer to as the “contactless probability” ($<P_{free}>$). We estimate this probability by counting, for each time step $t_k$, the number of particles that are not in contact with any other particles ($n_{free}(t_k)$) and dividing it by the total number of particles in the measurement area ($n(t_k)$). We then average these calculations over all recorded simulation time steps $t_n$, as shown by Eq. (5).

5 $$\begin{aligned} <P_{free}> = \frac{1}{t_n}\sum _{t_k=1}^{t_n} \frac{n_{free}(t_k)}{n(t_k)} \end{aligned}$$

Similarly, using the notation of angle brackets for the time average, the bulk density $<\rho>$ is calculated from the density in each time step $\rho (t_k) = n(t_k)/(16 \times 28)$, where the denominator represents the size of the measurement area.

Note that the number of particles in contact in each time frame is given by $n_c(t_k)=n(t_k)-n_{free}(t_k)$. We also analyse the time average of this quantity $<n_c>$.

Finally, we calculate the mean overlap in each time step $t_k$, taking into account only the particles in contact, using the expression $\xi _c(t_k) = 1/n_c(t_k) \sum _{i=1}^{n_c} \xi _{ij}$, where $\xi _{ij}$ represents the overlap between all pairs of particles *ij* that are in contact within the measurement area. Then, the time average is obtained by computing $<\xi _c>$.

**Figure 5:**
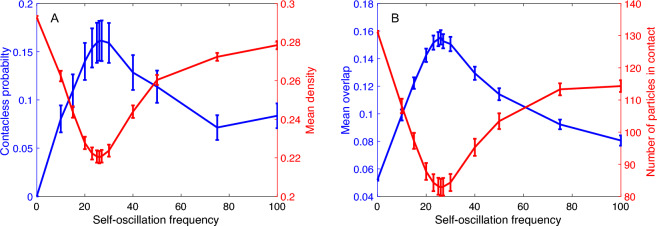
Observables (see text) are shown for the case where $k_n=500$, $v_f=3.3$, and $A=0.15$. In all cases, error bars represent one standard deviation from the time average. A: Contactless probability and mean density as a function of the self-oscillating frequency. B: Mean overlap between contacting particles and their quantity.

The quantities defined above are all displayed in Fig. 5.

As shown in Fig. 5A, the mean contactless probability and mean density vary as a function of the self-oscillation frequency. Notably, the contactless probabilities for all frequencies are less than 0.18, indicating that most particles are in contact with each other. The contactless probability is inversely related to the density, exhibiting a peak at the optimal frequency ($\omega ^*$). This analysis suggests that the flow rate is maximized when the particles have the fewest possible contacts, which can be achieved at minimum density.

In Fig. 5B, the mean number of particles in contact is explicitly shown along with the mean overlap for different values of $\omega$. Since the number of contacting particles is complementary to the number of free particles, it is natural to expect a minimum when the fraction of free particles $<P_{free}>$ has a maximum. However, it is interesting to observe that the mean overlap also displays a maximum at $\omega ^*$, indicating that even though there are fewer particles in contact, these contacts produce deeper overlaps, increasing the normal forces and consequently the repulsion energy, which causes a reduction in the density.

In summary, when the active oscillation of particles is at resonance with the natural frequency of their linear interaction, (a) the amplitude of these interactions is higher, which increases the overlaps and thus the repulsion energy, resulting in (b) a reduction in bulk density. The former suggests that particles can more easily pass through constrictions, particularly the bottom orifice, while the latter is indicative of a more fluid state in the bulk. These mechanisms allow for greater displacements and flow rates to be achieved.

## Discussion

Inspired by crowded cell systems, which consist of agents that actively change their shape, we propose an idealized model of self-propelled particles interacting via linear elastic forces and friction, with an active oscillation of their radii. This simple model is designed not to represent cell movement realistically but instead to investigate the effects of active deformation in the simplest possible system.

In our idealized system, we observe that particle deformation facilitates flow under conditions where the same particles would be unable to flow without deformation. This phenomenon occurs when the amplitude and frequency of self-oscillations exceed certain threshold values. Furthermore, we have identified an optimal frequency value ($\omega ^*$) that maximizes the flow rate. This self-oscillation frequency corresponds to the natural frequency of the system, which is determined by the linear stiffness coefficient of the particle interactions ($\omega ^* \sim \sqrt{k_n}$). To the best of our knowledge, this effect has not been previously reported.

We explain this behaviour in terms of the forced harmonic oscillations, in which the resonant frequency maximizes the amplitude of the oscillator. In our case, the oscillator pertains to the interaction between contacting particles. We determined that this maximization of amplitude correlates with maximum particle overlaps. Due to Eq. (3), this produces a maximum repulsion force, leading to a reduction in density and allowing a higher fraction of particles to be free of contacts. As a bulk effect, this maximizes the fluidization of the system. At the microscopic scale, maximum overlapping also leads to a minimum effective radius ($r_{eff}(t) < r_0$), generating a lower cross-section of particles, which allows them to occupy less space momentarily and increases the probability of passing through smaller spaces. This is in contrast to the case of very high frequencies, where the effective radius is approximately the mean particle radius ($r_{eff}(t) \sim r_0$).

We also found that the value of the optimum frequency ($\omega ^*$) is not influenced by the intensity of the self-propulsion mechanism, which is determined by the free velocity $v_f$. However, the corresponding maximum flow rate values exhibit the faster-is-slower effect.

The transition from clogging to flowing states when increasing the oscillation amplitude was also observed in experiments with vibrated silos^15,16,25^. Given this similarity, we can hypothesize that a similar response may be expected by tuning the oscillation frequency of the container. If this can be demonstrated, selecting an external vibration frequency that matches the natural frequency of the granular material could result in good flow rates at relatively low input energies.

Finally, our results provide an initial step towards building an active matter theory for crowds of cells that spans from simple to more complex model versions. Further investigation is needed to evaluate the effect of cell deformation on flow through narrow constrictions using models that are more realistic representations of cell motion, such as simple particles in the overdamped regime, or more complex simulation models that explicitly account for cell deformations, such as the cellular Potts model^30,31^.

## Supplementary Information

Supplementary Information 1.

## Data Availability

The datasets generated and analysed during the current study are available from the corresponding author upon reasonable request.

## Code availability

The source code associated with this research is publicly available and can be accessed^32^. The code repository contains the implementation of the algorithms described in this paper and can be used to reproduce the results.
