# Supplementary material for: Flow rate resonance of actively deforming particles
Source: Sci Rep. 2023 Jun 10;13:9455. doi: 10.1038/s41598-023-36182-5 (PMC10257709; doi:10.1038/s41598-023-36182-5)
Supplement: Supplementary file 1 — Supplementary Information 1. [file 41598_2023_36182_MOESM1_ESM.pdf]

Supplementary Methods for
"Flow rate resonance of actively deforming particles"

Value and error estimation for optimal self-oscillation frequency for different values of k_n

Consider the curves of self-oscillation frequency (ω) versus flow rate (Q) corresponding to different values of kn, as shown in Figure S1B. Each data points (ω_j ,$Q(\omega_j)$) can have different simulation durations (T_j) and therefore, different confidence levels.

The procedure for estimating the error bars for each point displayed in Fig. 4A of the manuscript is as follows.

1) For each curve depicted in Fig. S1B, the peak around the maximum value is approximated using a Gaussian function (f_g) that is fitted by finding the mean value (μ) and standard deviation (σ) that minimize the error weighted by the simulation duration:

$$E(\mu, \sigma) = \frac{\sum_{j=1}^{J} |f_g(\mu, \sigma, w_j) - Q(w_j)| \times T_j}{\sum_{j=1}^{J} T_j} \quad (1)$$

where,

$$f_g(\mu, \sigma, w_j) = \max(Q(\omega_i)) \exp(-1/2 \ (\omega_j - mu)/\sigma^2) \quad (2)$$

and $Q(\omega_i)$ are the flow rate values corresponding to all i data points of a given curve. The approximation given by equations (1) and (2) is also minimized by selecting the best J points around the maximum ($\max(Q(\omega_i))$.

2) Then, for each curve, we use the interpolated values inside the range given by J as shown in Fig. S1A. The optimum self-oscillation frequency (ω^*) is set to $\omega^* = \mu$.

3) Finally, to estimate the error, we take the width of the interpolated Gaussian function at $Q = 0.97 \ \max(Q(\omega_i))$, these widths are shown as horizontal solid lines in Fig. S1A, for each case.

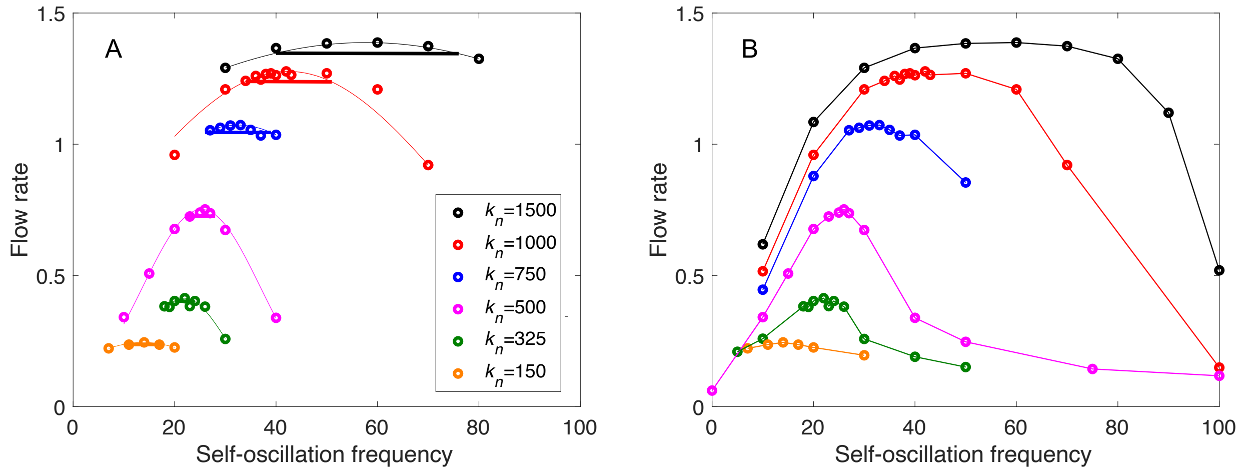

Figure S1: Estimation of ω^* and its error for different values of k_n using the reference values for the other parameters (see manuscript). Symbols are the result of a simulations with $T \, \varepsilon$ [5000, 25000].
